# On the Use of Monopole Antennas for Determining the Effect of the Enclosure of a Power Transformer Tank in Partial Discharges Electromagnetic Propagation

**DOI:** 10.3390/s16020148

**Published:** 2016-01-25

**Authors:** Ricardo Albarracín, Jorge Alfredo Ardila-Rey, Abdullahi Abubakar Mas’ud

**Affiliations:** 1 Generation and Distribution Network Area. Department of Electrical Engineering. Leader in Innovation, Technology and R&D, Boslan Engineering and Consulting S.A., Madrid 28034, Spain; rasbarracin@gmail.com; 2 Department of Electrical Engineering, Universidad Técnica Federico Santa María, Santiago de Chile 8940000, Chile; jorge.ardila@usm.cl; 3 Department of Electrical and Electronic Engineering Technology, Jubail Industrial College, Road No. 6, 8244 Al Huwailat, Al Jubail 35718, Saudi Arabia; abdullahi.masud@gmail.com

**Keywords:** power transformer tank, VHF and UHF sensors, monopole antennas, partial discharges, condition monitoring

## Abstract

A well-defined condition-monitoring for power transformers is key to implementing a correct condition-based maintenance (CBM). In this regard, partial discharges (PD) measurement and its analysis allows to carry out on-line maintenance following the standards IEC-60270 and IEC-60076. However, new PD measurements techniques, such as acoustics or electromagnetic (EM) acquisitions using ultra-high-frequency (UHF) sensors are being taken into account, IEC-62478. PD measurements with antennas and the effect of their EM propagation in power transformer tanks is an open research topic that is considered in this paper. In this sense, an empty tank model is studied as a rectangular cavity and their resonances are calculated and compared with their measurement with a network analyser. Besides, two low cost improved monopole antennas deployed inside and outside of the tank model capture background noise and PD pulses in three different test objects (Nomex, twisted pair and insulator). The average spectrum of them are compared and can be found that mainly, the antenna frequency response, the frequency content distribution depending on the PD source and the enclosure resonances modes are the main factors to be considered in PD acquisitions with these sensors. Finally, with this set-up, it is possible to measure PD activity inside the tank from outside.

## 1. Introduction

Measurement of partial discharges using very-high frequency (VHF), 30–300 MHz, and ultra-high frequency, 300–3000 MHz, sensors has become an important tool when monitoring and assessing the state of the insulation system on gas-insulated substations (GIS) and power transformers [1,2]. The current pulses that occur as a consequence of PD activity within this electrical equipment can generate the presence of electromagnetic emissions with bandwidths ranging from MHz to several GHz [3]. All information contained in these frequency bands can be captured and stored by using wideband sensors (antennas) which also does not require galvanic contact with any terminal of the equipment under test [4,5,6,7].

Besides, one of the main drawbacks when using externally such sensors in electrical equipment with self-shielding, according with its constructive nature, is that it may modify and/or mitigate the EM generated by PD activity inside the enclosure [8]. This happens in the case of power transformers, where part of the insulation system susceptible to the presence of PD is within this physical structure. In this regard, some studies have been addressed in order to establish the effect of shielding in the attenuation suffered by EM waves when pulses from inside are acquired from outside the transformer tank [8,9]. However, these papers have not taken into account the design of the sensor, and the resonance frequencies of the transformer tank, which vary according to the geometry and size of the structure [10], and the activity of different PD sources inside and outside the power tank.

In this paper, resonant wire antennas are justifiably selected to study the frequency behaviour of a transformer tank model when measuring PD, both from outside and inside the enclosure. With this aim, sequentially, test objects generating internal PD and surface PD inside the tank (vacuole in Nomex and twisted pair) and surface PD outside the tank (insulator) are acquired from antennas deployed inside and outside the enclosure to study their emissions in the UHF range. With post-processing and analysis of the measurements it will be possible to identify the main effects of a rectangular enclosure in PD propagation through a shielding such as a tank of a power transformer, which it contributes to a better characterization of the type of sources in this type of electrical equipment.

## 2. Selection of Antennas

To select accurately an antenna to measure EM emissions due to PD activity, it is necessary to know the main parameters that define its behaviour. Some of the most important are: *Directivity*, *Gain*, Bandwidth and *Scattering parameters* or *S*-parameters. Being $S_{11}=\frac{\left(Z_{a}-Z_{0}\right)}{\left(Z_{a}+Z_{0}\right)}$ the reflection coefficient, that indicates how much power is reflected from the sensor (frequency characteristic), where $Z_{a}$ is the load input impedance of the antenna and $Z_{0}$ is the 50 Ω impedance from the acquisition system. And the transmission parameter $S_{21}$, to obtain the frequency response of an emitter-transmitter radiofrequency (RF) system [11,12].

As it is known, disc couplers are the most common RF sensors for PD measurements in GIS and they are being slightly used in research models of power transformers [13]. However, the absence in the High-Voltage (HV) laboratory of a real power transformer including these sensors in which carry out the measurements, makes as requirement the use of a model of a transformer tank. A disc sensor has much sensitive response than a monopole antenna. However, the aim of the paper in this regard is to find an economical, alternative and easy to be implemented design antenna model to test with it. For this reason, the authors use low cost antennas such as monopoles due to their easy manufacturing. These kinds of antennas have been used in research for PD measurements such as in transformers [14,15], as well as, in GIS substation models [16] and inverters feeding electrical motors [17].

According to the antenna theory and design [12,18], the length of the antenna defines its main frequency resonance. Besides, a monopole antenna is half of a dipole antenna, thus, the definition of its first resonance is depicted as the Equation (1) and its first resonance is at λ/4.

(1) $$f_{r}=\frac{c}{l4}$$

where $\lambda =c/f_{r}$ and *l* is the length of the antenna. Being *c* the speed of light, $3\times 10^{8}$ ms${}^{-1}$, and $f_{r}$ the resonance frequency of the antenna in Hz.

Besides, the resonances for a monopole, given from the first one (λ/4) occur for λ/2, 3λ/4 and *λ*, according to [12].

With the aim of selecting the best configuration of a monopole, in this paper four lengths are analysed and their theoretical results are shown in Table 1. The requirements for the antenna selection were a suitable frequency response with the smallest possible length to avoid electrical hazards.

**Table 1:** Theoretical resonance frequencies for monopole antennas.

Monopole antenna	$f_{r}$(λ/4) (MHz)	$f_{r}$(λ/2) (MHz)	$f_{r}$(3λ/4) (MHz)	$f_{r}$(*λ*) (MHz)
**1 cm**	7500	15000	22500	30000
**5 cm**	1500	3000	4500	6000
**10 cm**	750	1500	2250	3000
**16.5 cm**	454	910	1364	1820

As described in experimental setup (Section 5), the measurement equipment used can reach up to 2.5 GHz, so the resonances that can be measured are gray as marked in Table 1. The frequency resonances for the 1 cm monopole cannot be measured, so its used is discarded. In addition, the oscilloscope only can represent the first resonance of the 5 cm monopole, thus this antenna is also rejected. With respect to the 16.5 cm monopole, although it has their frequency resonances into the analysed bandwidth, its high length can produce electric hazard due to contact with the feeding of the setup or with energized electrode of the test object. Moreover, this drawback can be increased in real power transformers with the possibility of electrical contact with their windings, so this sensor is also discarded. Thus, the length of the monopole elected is 10 cm, even more, it has its three first resonances below than 2.5 GHz band, so it could measure energy from PD with less risk of electric hazard than the 16.5 cm monopole.

An Agilent E8364B network analyser (NA) is used to measure the frequency response ($S_{11}$ parameter) for the monopole antenna. Likewise, this kind of resonant antennas could be improved by introducing a ground plane at its terminal so that it works as dipole antenna. Thus, to evaluate the performance of the sensor, the measurements with the NA was carried out with and without a ground plane. The main advantage of a dipole antenna is to have the double gain and directivity than a monopole antenna with the same length. Thus, for a λ/4 dipole its gain is $G_{\frac{\lambda }{4}}≃e_{cd}3.286$ dB and its directivity $D_{\frac{\lambda }{4}}=5.167$ dB, only applicable for their resonance frequencies at *λ*/4, *λ*/2, 3*λ*/4 and *λ*. Where $e_{cd}$ is the radiation efficiency of the antenna, being $e_{cd}≃1$ at the frequency resonances of monopoles and dipoles sensors [12,18].

In Table 2, it is shown the resonance frequency values for the 10 cm antenna with and without a ground plane together with the relative error (RE) between the theoretical value and the experimental measure. Note that a *λ*/4 monopole or dipole antenna has the same theoretical frequency resonances, so the comparison between the results of calculations for the monopole (dipole) can be carry out with those obtained from measurements on dipole. Besides, it can be observed that the match between the resonances calculated theoretically, Table 1, and the experimental results using a ground plane is better than those obtained without the ground plane, Table 2. In addition, when a monopole or dipole type antenna is used, sometimes, the resonant frequencies are shifted with respect to the theoretical ones [11].

In this case, the dipole antenna has a better match of the frequency resonances with the theoretical calculation made in Table 2, with an error between [−15,+15]%. These results are contrasted with those obtained in [11], where the frequency response of a 10 cm monopole antenna with and without ground plane is quite similar, however the power spectrum is greater for the dipole antenna than that obtained at their analogous resonant frequencies (approximately at 750 MHz and 1500 MHz) for the monopole.

**Figure 1:**
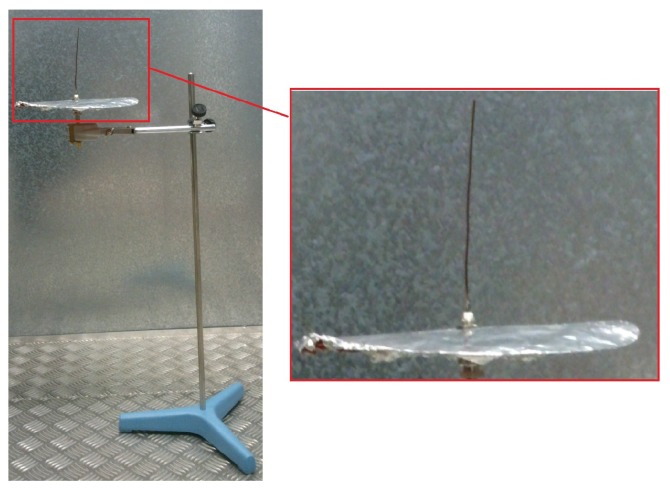
10 cm monopole with ground plane.

In Table 2, RE below than 15% are gray marked. Negative values mean that the theoretical value is below than the experimental one, while a positive number is interpreted as the theoretical frequency has a value above the measure. The 10 cm monopole with ground plane has a RE below than 15% for all the resonance frequencies measured, while a RE above 29% is obtained for the same antenna without ground plane. Thus, a 10 cm monopole with ground plane dipole antenna sustained with a tripod, as in Figure 1, is selected for the measurements.

**Table 2:** Experimental values of the resonance frequencies of the 10 cm antenna working as dipole or monopole antenna for *λ*/4, *λ*/2, 3*λ*/4 and *λ*.

		$f_{r}$(*λ*/4)		$f_{r}$(*λ*/2)		$f_{r}$(3*λ*/4)		$f_{r}$(*λ*)	
**Antenna**		**Monopole**	**Dipole**	**Monopole**	**Dipole**	**Monopole**	**Dipole**	**Monopole**	**Dipole**
10 cm	MHz	530	640	1060	1730	1385	2150	2137	2600
	%	29	15	29	−15	38	4	29	13

## 3. Power Transformer Tank

In order to have a representative model of a real enclosure for an oil-immersed power transformer, [19], a 30 × 30 × 50 cm tank made of steel plates of 5 mm thickness are constructed to carry out PD measurements in RF. The material and thickness are selected according to those used in a power transformer 15 kV/420 V of 25 kVA deployed in distribution networks.

**Figure 2:**
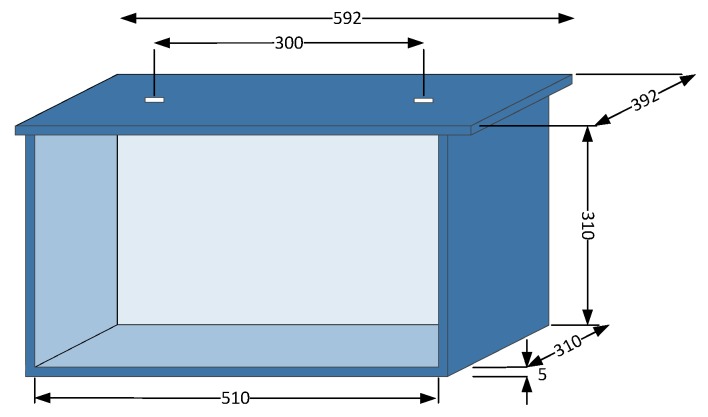
Dimensions of the tank, in mm.

For a first approximation, this scale construction is suitable to reproduce the same electromagnetic effects that can be found in an empty transformer tank. The upper cover has two entrances separated 30 cm, which allow one, the passage of the high-voltage electrode in the tank to feed the indoor test object and the other input for an antenna deployment. These holes are 3 cm in diameter and are protected by a Teflon hollow cylinder serves to prevent electrical contact between the housing, grounded, and the test object that is placed inside the tank. Finally, a cork layer is used as a mechanical fit between the tank and the cover, just as in distribution transformer tanks. The Teflon hollow cylinder and the layer between metal and cork acts as dielectric windows for signals in RF. The model is represented in Figure 2 with the front wall erased for a better understanding.

In next subsections, it is shown the behaviour of the power transformer tank implemented considered as rectangular resonant cavity, their theoretical frequency resonances and their measurements when PD activity it is taken into account.

### The Tank as a Rectangular Resonant Cavity

A transformer tank is a rectangular structure that has an electromagnetic behaviour as a resonant cavity [12]. So, when there is PD activity inside the cavity, multiple reflections create stationary waves between its conductive metal walls which generate resonance frequencies. These stationary waves are the transverse magnetic (TM) and transverse electric (TE). TM has no components of magnetic fields in the direction of propagation $H_{z}$ = 0, but being nonzero its electric field component, $E_{z}$. While, TE wave, has no components of the electric field in the direction of propagation $E_{z}$ = 0, but with nonzero components of magnetic field, $H_{z}$.

To designate the distribution of a stationary wave TM and TE for axis *x*, *y*, *z* of a resonant cavity, subindex *mnp*, are used. For the calculation of these modes, the following equation is used [10,20]:

(2) $${\left(f_{rc}\right)}_{mnp}=\frac{1}{2\pi \sqrt[{}]{\mu \epsilon }}\sqrt[{}]{{\left.\left(\frac{m\pi }{a}\right)\right.}^{2}+{\left.\left(\frac{n\pi }{b}\right)\right.}^{2}+{\left.\left(\frac{p\pi }{c}\right)\right.}^{2}}$$

where $f_{rc}$ is the resonance frequency of the cavity, $\mu =4\pi \times 10^{-7}$ Hm/A the permeability of vacuum, $\epsilon =8.85\times 10^{-12}$ F/m the permittivity of vacuum and *a*, *b*, *c* the dimensions of the tank in *m*.

Equation (2) is used to calculate the frequencies in which the rectangular cavity resonates and to compared these frequencies with their measurement in next subsection.

Assuming that the maximum length of a transformer tank corresponds to the *z*-axis propagation, width with the *x*-axis and height with the *y*-axis, then it is possible to calculate the results of the resonance frequencies ${\left(f_{rc}\right)}_{mnp}$, according to Equation (2), for transverse electric modes, TE${}_{mnp}$, and transverse magnetic, TM${}_{mnp}$. This equation is applied to the tank geometry studied for calculating their resonant frequencies. The first resonance frequency is obtained at 583 MHz.

**Figure 3:**
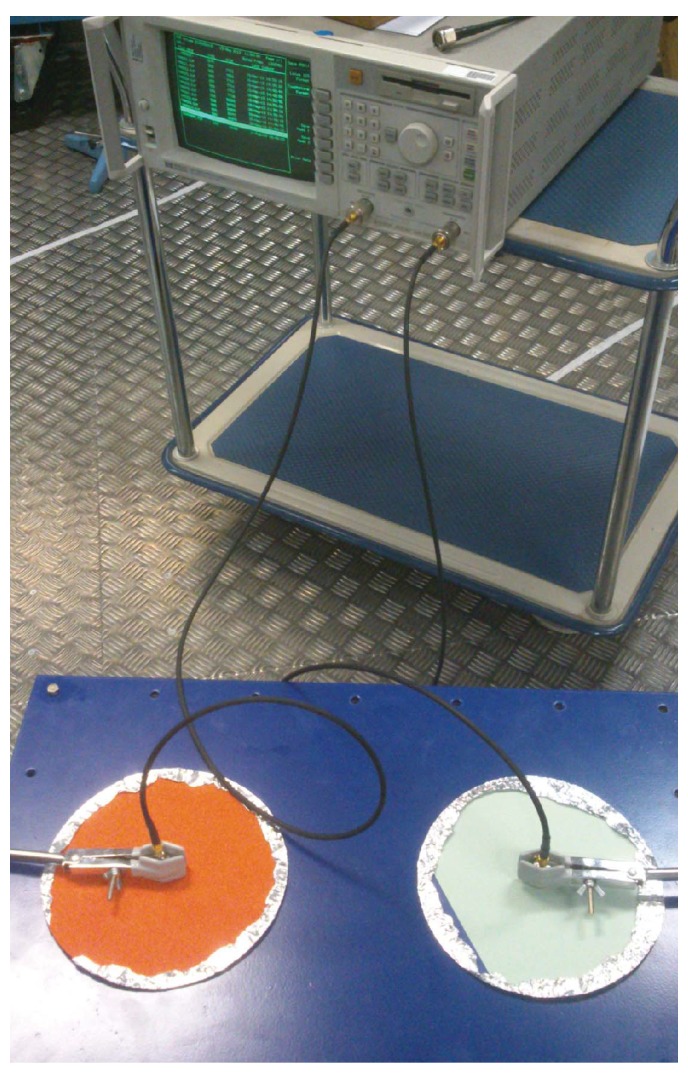
Two 10 cm monopole antennas with ground plane and an Agilent E8364BD network analyser for the measurement of the $S_{21}$ parameter of the tank.

To have a measure of these frequencies to be compare with its theoretical calculations, the transmission parameter $S_{21}$ is measure to obtain the frequency response of the tank. For the acquisitions, the Agilent E8364B network analyser and two antennas are used, thus, one injects energy in the tank by sweeping the frequency of the NA between 500 MHz and 2500 MHz and the other acts as a receiver. Figure 3 shows the positions of the antennas deployed inside the tank through the cavities on the top cover.

**Figure 4:**
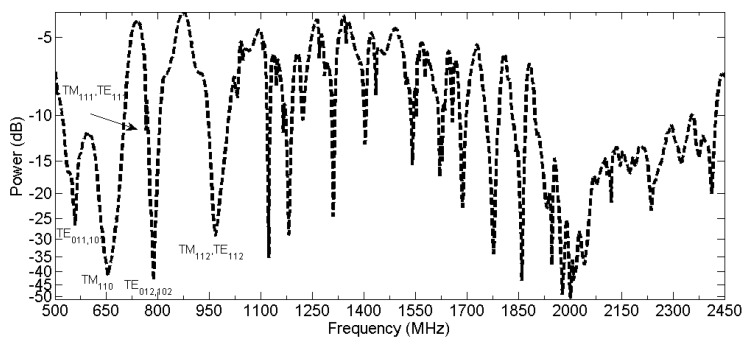
$S_{21}$ parameter (frequency response of the tank) measured with two 10 cm dipoles.

In Figure 4 the $S_{21}$ parameter measured with two 10 cm monopoles with ground plane together with the network analyser is shown.

The calculated frequency resonances, their modes, the resonances measured ${\left(f_{rc}\right)}_{mnp}$ (MHz), their power (dB) and the ER between frequencies calculated and measured are shown in Table 3 up to 2000 MHz. When there is no mode or no measurement of a resonance frequency it is represented by a dash (−) in Table 3.

**Table 3:** Frequency resonances for a 30 × 30 × 50 cm cavity in 500–2000 MHz.

${\left(f_{rc}\right)}_{mnp}$ (MHz) Calculated	TM ${}_{mnp}$	TE ${}_{mnp}$	${\left(f_{rc}\right)}_{mnp}$ (MHz) Measured	Power (dB)	RE (%)	${\left(f_{rc}\right)}_{mnp}$ (MHz) Calculated	TM ${}_{mnp}$	TE ${}_{mnp}$	${\left(f_{rc}\right)}_{mnp}$ (MHz) Measured	Power(dB)	RE (%)
583	−	011, 101	560	−26	3.89	1535	222	222	1540	−15	−0.31
707	110	−	655	−41	7.30	1561	−	024, 204	1552, 1560	−10, −6	0.57, 0.06
768	111	111	765	−11	0.34	1580	130, 310	015, 105	1578, 1590	−7, −6	0.13, −0.63
780	−	012, 102	787	−43	−0.83	1608	131, 311	131, 311	1602	−8	0.39
927	112	112	967	−29	−4.35	1614	032, 302	−	1620	−17	−0.34
1029	−	013, 103	1032	−8	−0.31	1639	124, 214	124, 214	1628, 1635	−15, −10	0.67, 0.24
1043	−	021, 201	1048	−6	−0.45	1657	115	115	1658	−11	−0.05
1117	120, 210	−	1105, 1122	−6, −35	1.10, −0.42	1675	225	225	1665	−6	0.61
1144	113	113	1145	−8	−0.11	1690	132, 312	132, 312	1688	−23	0.12
1157	121, 211	121, 211	1165	−12	−0.71	1748	−	033, 303	1778	−34	−1.71
1165	−	022, 202	1180, 1222	−29, −10	−1.25, −4.86	1802	230, 320	025, 205	−	−	−
1268	122, 212	122, 212	1270	−6	−0.16	1818	133, 313	133, 313	−	−	−
1299	−	014, 104	1285, 1310	−7, −24	1.09, −0.84	1826	231, 321	231, 321	1842	−14	−0.86
1344	−	023, 203	1348	−5	−0.27	1853	224	224	1842	−14	0.62
1392	114	114	1402	−13	−0.73	1867	−	016, 106	1860	−43	0.37
1413	220	−	−	−	−	1870	125, 215	125, 215	−	−	−
1434	123, 213	123, 213	1435	−8	−0.05	1899	232, 322	232, 322	1902	−12	−0.17
1445	221	221	−	−	−	1920	−	034, 304	−	−	−
1529	−	031, 301	1522	−10	0.43	1933	116	116	1932	−23	−0.03

Experimentally, almost all modes calculated theoretically are obtained, except at high-frequencies, where resonances are not reproduced.

The dominant resonance mode corresponds to TE${}_{011}$ and TE${}_{101}$. The first resonance for the dominant transverse mode corresponds to TM${}_{110}$. In this cavity, these modes are reproduced at 583 MHz and 707 MHz, TE and TM, respectively. When they are measured with the network analyser, the first TE mode is obtained at 560 MHz with a RE of 3.89%, compared with the theoretical values, and -26 dB, where the negative value indicates emitted power inside the tank. Besides, the first TM mode has its resonance frequency at 655 MHz with a RE of 7.30% and power of -41 dB, this high energy value received, is because the antenna has its first resonance at 640 MHz as it is mentioned above.

As it is shown in Figure 4, experimentally, not all modes are excited in the resonant cavity for frequencies above 2300 MHz. Assuming that the network analyser emits the same power at all frequencies, then the receiving or transmitting antenna, are not able to excite the frequencies within the tank. However, for lower frequencies, the tank is capable of resonating in the most theoretically calculated modes in Table 3.

When the tank resonance modes are measured, there are mainly two factors that affect the result. First, the modes defined by the structure. To obtain these resonances, a frequency sweep is done with the network analyser up to 2500 MHz. The second is the frequency response of the antenna used, that must be matched for all the bandwidth required. However, the hole size to accommodate the antenna on the tank and its geometry do not allow to deploy an antenna that meets these requirements. By restriction of size, and to have a cheaper antenna than a disc-coupler to do the measures, a monopole antenna is used because can also be deployed inside the empty model tank through the holes and can acquire energy up to 2250 MHz for the 10 cm in length antenna.

In the experimental measurements with the transformer tank model, almost all their own theoretical frequencies of a rectangular resonant cavity of the same dimensions are obtained, as shown in Table 3 [10]. To study only the effect that the tank has on PD propagation, the tank model unfilled of oil and without placing a magnetic core and windings therein it is used for measuring discharge with the antennas. Inside, it is expected to measure the direct wave of the discharge and the pulses reflected in the shield walls that excite their resonant frequencies. Outside, it is intended to receive power content from frequencies with the higher energy, mitigated by the enclosure, which goes through the holes in the top and the joints between the cover and the walls. These components depend on the frequency response of the test object emitting and the resonance frequencies that allows the cavity.

## 4. Test Objects

To study the effect of the transformer tank in the RF propagation of the PD, three types of test objects are used and measurements are carried out sequentially to ensure repeatability. First, Nomex paper is located inside the tank ensuring that the activity from internal PD occurs inside the tank. The second test object generates surface PD in a twisted pair deployed into the tank. Finally, PD surface are measured on an insulator located on the tank cover.

**Figure 5:**
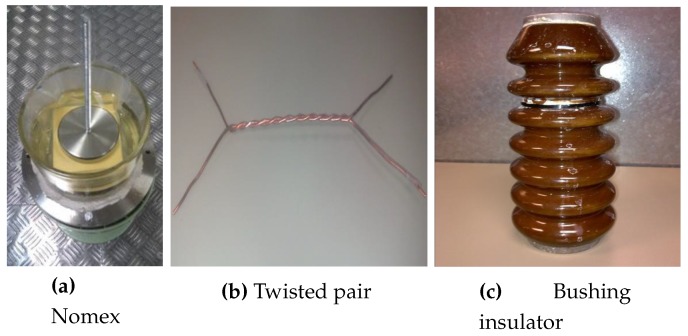
Test objects: (**a**) Nomex 4-3-4 submerged in oil, (**b**) twisted pair of copper wire type Pulse Shield SD, 23.5 × 12 cm with a central twist of 13.5 cm and (**c**) bushing insulator with an electrode in the top, 21 × 8 cm.
  Finally, it is used a bushing insulator made of porcelain commonly used in medium voltage transformers, Figure 5c. To obtain PD over its surface it is contaminated with saline and the measurements are carry out once moisture has disappeared. This kind of polluted environments are a very common situation in overhead lines in coastal areas. Some of the agents that pollute these elements are dust, bird droppings, rain, ice and, near the sea provisions, damp and saline environment. In locations with a high probability of contamination, the possible activity of this type of discharge can be critical and measures should be taken. In locations with a high-probability of contamination, the possible activity of this type of discharges can be critical and their measure should be taken into account.

- Internal PD are the most harmful for the insulation system and their detection is essential to carry out proper maintenance based on the condition of the electrical equipment [21,22]. Internal discharges occur in defects, holes, into the insulating systems with low dielectric strength. The accomplishment of vacuoles in a solid insulation can be a difficult task, for example in epoxy, due to requires raising its temperature and inject air bubbles through a needle. The main drawback is that it is not possible to control the geometry of the hole and, also the needle always leaves a return path in the material. As a solution to these problems, the idea of segmenting the solid insulation in several layers is used, drilling by a needle the intermediate sheet to generate a cylindrical imperfection in the insulation. Each of these layers must be cut it with the same shape to be fitted inside the oil vessel in which it is housed, and they are glued so that when performed the hole it is aligned. Finally, when the vacuole is done, the needle leaves a burr to be removed from the material.
  The test object for internal PD is housed in a glass vessel with a steel bases, that can be connected to ground. This vessel contains mineral dielectric oil Nytro Taurus, used in high-power transformers. Inside the vessel are 11 insulating layers and above them an electrode made of steel which connects a cable to apply voltage to the sample. Insulating layers are laminated flexible Nomex, F-20.08 Triplex electrical insulation manufacturer *Royal-Diamond*, Figure 5a. This material is a sheet coated on both sides with polyester fibres. In its practical application, is inserted in windings of transformers, motors and generators subjected to high-mechanical, dielectric and thermal requirements. Each of those 11 layers has a thickness of 0.35 mm, 3 core layers are perforated with a needle to create a small cylindrical cavity diameter of 1 mm and a length of 1.05 mm. In addition, the assembly is placed in a plastic bag and the air is removed with a vacuum machine. Thus, it is to ensure that this vacuole has the dielectric constant in vacuum that is lower than that of the insulating material. Therefore, internal PD can occur in the cavity at voltage levels relatively small.
- Surface PD appear between two dielectrics, usually between the insulation system and air. Pollution or moisture, e.g., in insulators, may accelerate the process fetterless of this type of discharge and can even lead to a short-circuit between the overhead line and the tower connected to ground. As another example, when PD are produced in the insulated phase feeding a power transformer, they can cause a fault between the input line and the tank, leading to equipment out of service.
  A twisted pair of copper wire type Pulse Shield SD and manufactured by Rea Magnet Wire Company is used Figure 5b. Its enamel consists of an insulating layer of resin polyamide-imide modified and an over layer of modified polyester (THEIC) trishydroxyethyl isocyanurate. The set has a thickness of 1 mm and can withstand temperatures up to 220 ${}^{\circ }$C with a lifetime of 20,000 h and voltage levels until failure 5.7–11 kV.

## 5. Experimental Setup

**Figure 6:**
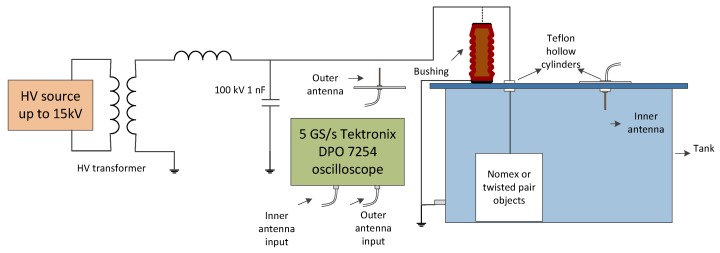
Experimental set-up scheme for the PD acquisitions for the three test objects.

At the very beginning, the type of discharges are previously confirmed using the commercial TechImp PD Check, its software PD Base and a High-Frequency Current Transformer (HFCT) with the indirect circuit, according to IEC-60270 standard [23]. For the measurements with the antennas, RG-223 coaxial cables 5 m long, with BNC connectors are used. The cables are connected to a Tektronix DPO 7254 oscilloscope 5 GS/s and the set-up is represented in Figure 6.

**Figure 7:**
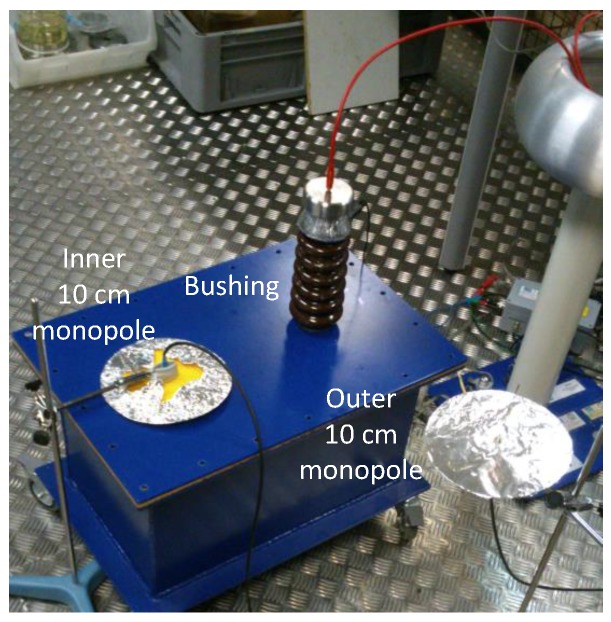
Layout of 10 cm monopole antennas with ground plane, inside and outside the tank, to measure surface PD from the bushing insulator at 12 kV.

For these tests, two antennas, one inside and one outside the tank are deployed to determine the influence of shielding in PD propagation both inside and outside. As is indicated in Section 2, two antennas of 10 cm in length with a ground plane will be used to have comparable sensors inside and outside. The antenna inside the tank is located 30 cm from the test object and the second is placed outside the tank at the same distance, to have the same time-delay between pulses. When the bushing insulator is used, it is placed over the tank and equidistant to the outer antenna, which is also located at 30 cm, to be consistent with the others’ experiments. Figure 7 shows the arrangement of the setup, which is the same for all experiments, of the antennas and the tank for measurements with the insulator. As it is shown, both antennas are supported by a tripod that prevents movement.

## 6. Experimental Measurements and Results

### 6.1. Influence of the Tank in PD Measurements with Antennas

The measurement campaigns are made with 50 pulse acquisitions at 5 GS/s with a time window of 0.5 *μ*s in the oscilloscope channels where the antennas are connected. For each pulse, the fast Fourier transform (FFT) was calculated using a rectangular window. Each frequency component n-th from the FFT was scaled to calculate the root-mean-square (RMS) and squared to obtain the power spectrum $P_{f_{n}}$, in $V^{2}$, of the signal at frequency $f_{n}$, being $A_{f_{n}}$ the FFT amplitude, Equation (3) [8].

(3) $$P_{f_{n}}=\left(\frac{{A_{f_{n}}}^{2}}{\sqrt{2}}\right)$$

In order to compare significant bands of frequency, several intervals are selected for each test object depending on the frequency components. Equation (4) is used to obtain the accumulative power in bands $\Delta P_{\left[f_{L},f_{H}\right]}$ in $V^{2}$ in various intervals between a lower and higher frequency values, $f_{L}$ and $f_{H}$, respectively.

(4) $$\Delta P_{\left[f_{L},f_{H}\right]}=\sum_{n=f_{L}}^{f_{H}}\left(\frac{{A_{f_{n}}}^{2}}{\sqrt{2}}\right)$$

Equation (5) is used to calculate the signal-to-noise ratio (SNR) for the accumulative power when PD are acquired and when no voltage is applied. When SNR > 1 then the background noise power is lower than the PD power in the $\left[f_{L},f_{H}\right]$ band.

(5) $$SNR=\left(\frac{\Delta P_{\left[f_{L},f_{H}\right]}^{PD}}{\Delta P_{\left[f_{L},f_{H}\right]}^{noise}}\right)$$

**Figure 8:**
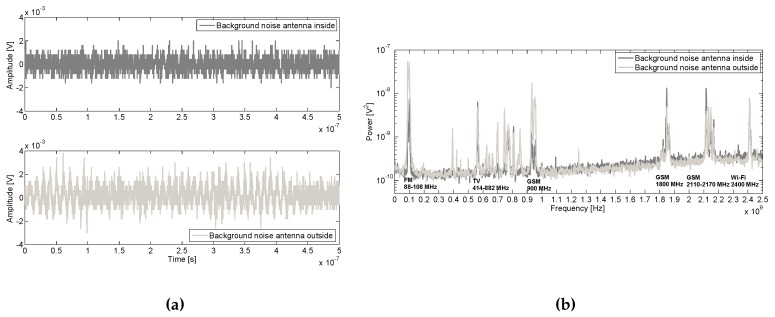
Background noise measured inside and outside the tank, (**a**) pulses and (**b**) spectrum with continuous sinusoidal noise from communications identified.

For the noise acquisition in the laboratory, no voltage was applied and the synchronization was performed with the AC 50 Hz line voltage of the oscilloscope. In Figure 8a a background noise pulse is represented for both inner and outer antennas. Additionally, the averaged spectrum of 50 pules of each antenna are compared in Figure 8b and the sinusoidal signals from communication systems such as:

- Modulated frequency (FM) radio
- Digital television (TV)
- Global System for Mobile communications (GSM)
- Wireless Fidelity (Wi-Fi)

are depicted. The frequency response inside the tank for FM, TV and GSM at 900 MHz are mitigated, however, the spectrum power is quite similar for both for GSM at 1800 MHz, 2110−2170 MHz and Wi-Fi sources.

**Figure 9:**
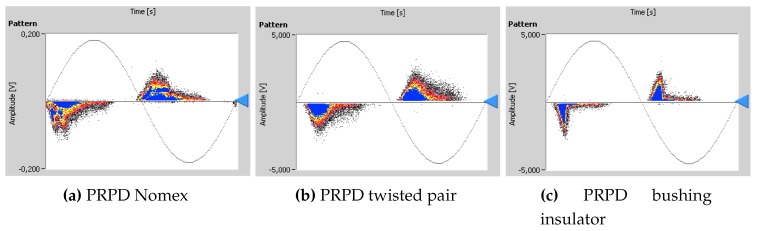
PRPD patterns: (**a**) Internal PD in Nomex 4-3-4 submerged in oil, (**b**) surface PD in twisted pair and (**c**) surface PD in bushing insulator.

Once the pulses from noise have been acquired, then the PD activity for each test object was measured. The voltage level was raised above the voltage ignition, $v_{i}$, for which PD activity start, up to set the applied voltage, $v_{a}$, Table 4. The measurement was carried out after 10 min to ensure a stable emission activity of PD and with a trigger level slightly above the noise level. The Phase-Resolved PD (PRPD) pattern for each PD source is registered as explained in the previous section and they are represented in Figure 9.

**Table 4:** Ignition and applied voltages for the test objects used.

Test Object	$v_{i}$ (kV)	$v_{a}$ (kV)
Nomex	9	13
Twisted pair	0.65	0.76
Porcelain insulator	10	12

### 6.2. Internal PD in Nomex Inside the Tank

**Figure 10:**
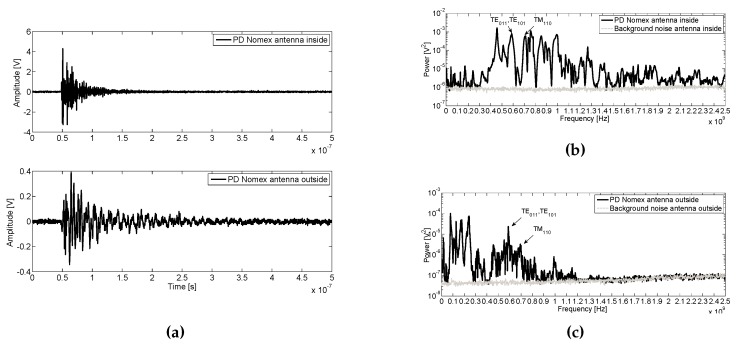
(**a**) Internal PD pulses in Nomex paper at 13 kV inside the enclosure acquired by the inner and outer antennas and their average power spectra compared with the noise inside (**b**) and outside (**c**).

Figure 10a shows an internal PD pulse for Nomex measured by both antennas. Their spectrum compared to background noise are depicted in Figure 10b,c, inside and outside the tank, respectively. As might be expected, the signal for the inner antenna has more components in UHF than those measured outside the enclosure, which are mitigated due to the lost of its energy when the pulse is rebounding inside the tank [24].

As it is shown in Figure 10b,c, there is a power increase below the frequency of the first resonance mode TE${}_{011}$, TE${}_{101}$ = 560 MHz, mainly at 450 MHz. All these components of the spectrum are due to the characteristics of the direct emission radiated of this type of internal PD at these frequencies [8], the frequency response of the antenna and the resonance characteristic of the enclosure.

For the power spectrum calculated for the pulses acquired with the antenna outside the tank, it can be seen that no significant power is received from the PD above 1.2 GHz, so the tank acts as a low pass filter. Furthermore, the content of power at 450 MHz is also received outside and mainly up to 400 MHz. Indeed, more power below 400 MHz is received outside the tank than inside. This can be due to the cable to ground of the enclosure which act as an antenna whose length radiate in these frequency ranges when the PD leave the cavity conductively through the ground path.

Finally, the first two resonance modes are marked, TE${}_{011}$, TE${}_{101}$ = 560 MHz and TM${}_{110}$ = 655 MHz in Figure 10b,c verifying that they can be measured from outside the tank under these circumstances. Table 5 compares the first resonance modes for Nomex inside and outside the tank. As can be expected, in both cases, the peak power values are greater inside than outside. To compare which has the highest magnitude, the power inside is divided by the power outside (Ratio), so values greater than 1 mean that the mode has higher power inside the tank and viceversa.

**Table 5:** Peak values for first TE and TM modes inside and outside the tank for internal PD in Nomex.

Modes	Power ($V^{2}$)		
	Inside	Outside	Ratio
${\mathrm{TE}}_{011}$, ${\mathrm{TE}}_{101}$ = 560 MHz	8.1 × $10^{-4}$	5.9 × $10^{-4}$	1.4
${\mathrm{TM}}_{110}$ = 655 MHz	2.4 × $10^{-5}$	3.3 × $10^{-6}$	7.3

Table 6 presents the accumulated power measured with the inner and outer 10 cm monopoles. In this case, three bands are analysed with their SNR: up to 300 MHz where there is the main contribution in power from the outer antenna; 300–1200 MHz, where there are the first resonance modes and; 1200–2500 MHz, where no PD radiation is received from outside. Focused on the 300–1200 MHz band, the ratio for the inner antenna is 166.5 while outside is 35.5, so it could be measured the the resonances inside the tank from outside with a high SNR. In Table 6, also the two first modes are included as example of comparison, with high values for the measure inside the tank. Up to 300 MHz, the ratio outside 214.3 is much greater than inside 2.6, so this components are not coming from inside the enclosure. From 1200–2500, the power is greater inside with a ratio of 6.8 than outside, 1.1, thus their low power can not come out the enclosure.

**Table 6:** Accumulated power and SNR for internal PD in Nomex inside the tank when measured with the inner and outer monopoles of 10 cm.

Antenna	Power ($V^{2}$)		
	13 kV	0 V	SNR
Inside ∼300 MHz	3.4 × $10^{-4}$	1.3 × $10^{-4}$	2.6
Outside ∼300 MHz	1.4 × $10^{-3}$	6.3 × $10^{-6}$	214.3
Inside 300–1200 MHz	6.6 × $10^{-2}$	4 × $10^{-4}$	166.5
Outside 300–1200 MHz	8.3 × $10^{-4}$	2.3 × $10^{-5}$	35.5
Inside 1200–2500 MHz	4.4 × $10^{-3}$	6.4 × $10^{-4}$	6.8
Outside 1200–2500 MHz	5.4 × $10^{-5}$	4.9 × $10^{-6}$	1.1

### 6.3. Surface PD in a Twisted Pair Inside the Tank

**Figure 11:**
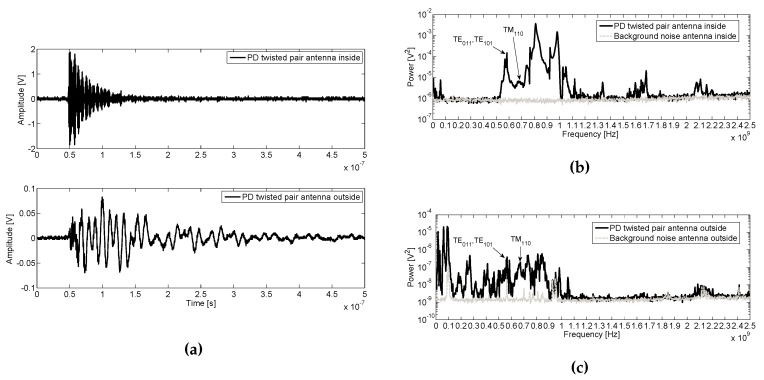
(**a**) Surface PD pulses in twisted pair at 760 V inside the enclosure acquired by the inner and outer antennas and their average power spectra compared with noise inside (**b**) and outside (**c**).

In Figure 11a surface PD pulses with the twisted pair inside the tank are shown from inside and outside. For the inner antenna, the pulse has high amplitude than outside the enclosure.

The spectrum of noise compare with PD obtained with both antennas are shown in Figure 11b,c. Inside the tank, the power is concentrated mainly between 500–1100 MHz with a maximum high-power peak value of 3 $\times 10^{-3}$ $V^{2}$. First resonance modes are marked on figures for these frequencies. Besides, it can be shown that there are other frequency components in two distinguished bands, the first up to 100 MHz and, the second, above 1100 MHz. In the second band, the power is concentrated between 1100–1400 MHz, 1500–1800 MHz and at 2100 MHz, with a peak value of 3 $\times 10^{-3}$ $V^{2}$ at 1700 MHz. As in the previous experiment, the power spectrum of the PD outside the tank is not increased above 1.2 GHz, receiving only the GSM and Wi-Fi emissions at 2.1 GHz and 2.4 GHz, respectively. Besides, an increase in power for the range of 500–1100 MHz due to PD activity in the tank is observed. Up to 150 MHz, there is a power increase from PD with a similar maximum power level as inside, taking values of $10^{-5}$ $V^{2}$. Note that, in the 150–500 MHz range, there is a power increase outside the tank that is not shown from inside due to the different scales used. This is because the pulse inside is 20 times greater than outside the tank, thus the high-scale needed to represent the inside pulse not allows enough resolution to identify low power components in the order of $10^{-6}$ $V^{2}$ because its peak power is at $5\times 10^{-3}$ $V^{2}$.

In Table 7 the first resonance modes for twisted par test object measured inside and outside the tank are compared. As expected, for both, the peak power values are greater inside than outside.

**Table 7:** Peak values for first TE and TM modes inside and outside the tank for surface PD in twisted pair.

Modes	Power ($V^{2}$)		
	Inside	Outside	Ratio
${\mathrm{TE}}_{011}$,${\mathrm{TE}}_{101}$ = 560 MHz	1.5 × $10^{-4}$	6.6 × $10^{-6}$	22.7
${\mathrm{TM}}_{110}$ = 655 MHz	3.8 × $10^{-7}$	1.9 × $10^{-7}$	2

Table 8 presents the accumulative power measured with the 10 cm monopoles. The three more significant intervals are: up to 500 MHz, where power from PD is seen outside and not from inside; 500–1200 MHz; and 1200–2500 MHz. Note that the intervals analysed are different within experiments depending on the frequency content nature in each test object. In this case, inside, the most representative band of power is 500–1200 MHz with a ratio of 158.6 which it is also measured from outside with a good SNR of 29.1 making it possible to measure the power content of the first resonances of the tank, excited by this type of PD, from outside and with a good SNR. Thus, discriminating the bands in which the kind of PD emits power, it is possible discern, in this case, if there is a concrete kind of discharges into the tank. Outside, the power contents of the first band up to 500 MHz has a higher SNR, 186.3, however this effect is only representative up to 100 MHz from inside Figure 11b. From 1.2 GHz, the tank do not allow to leave out radiation from inside.

**Table 8:** Accumulated power and SNR for surface PD in twisted pair inside the tank when measured with the inner and outer monopoles of 10 cm.

Antenna	Power ($V^{2}$)		
	760 V	0 V	SNR
Inside ∼500 MHz	2.6 × $10^{-4}$	2.1 × $10^{-4}$	1.2
Outside ∼500 MHz	1.7 × $10^{-4}$	9 × $10^{-7}$	186.3
Inside 500–1200 MHz	5.1 × $10^{-2}$	3.2· $10^{-4}$	158.6
Outside 500–1200 MHz	2.1 × $10^{-5}$	7.3 × $10^{-7}$	29.1
Inside 1200–2500 MHz	1.1 × $10^{-3}$	6.9 × $10^{-4}$	1.7
Outside 1200–2500 MHz	1.5 × $10^{-6}$	1.2 × $10^{-6}$	1.3

### 6.4. Surface PD in an Insulator on the Tank Cover

**Figure 12:**
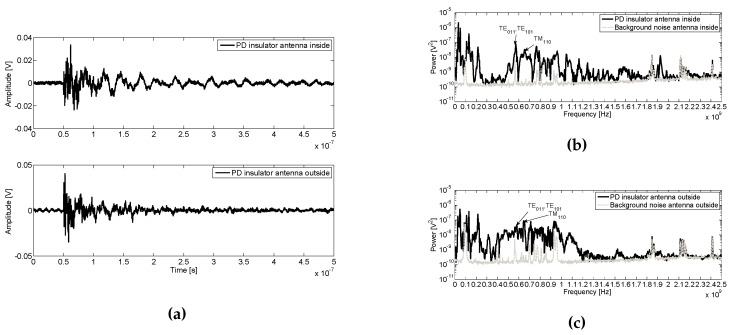
(**a**) Surface PD pulses on insulator at 12 kV outside the enclosure acquired by the inner and outer antennas and their average power spectra compared with the noise inside (**b**) and outside (**c**) the enclosure.

Figure 12a shows pulses for surface PD on the insulator supported on the cover tank. Spectra of noise and PD are represented in Figure 12b,c from inside and outside the enclosure, respectively. Inside and outside, this type of PD have a frequency distribution with a large content of power up to 300 MHz and between 300–1200 MHz as can be seen in, Figure 12b,c and in this second band is precisely where the first tank resonant modes are. It should be expected that external signals can not be measured inside the tank, but this is not so, and the distribution of power measured inside and outside is quite similar. Into the tank, resonances are observed and their power content is amplified for values above 1200 MHz. Below 300 MHz, the power is markedly amplified inside the tank compare to outside. This may be because the antenna is located in the hole of the cavity and the insulator above the tank, so the sensor can receive the pulse directly, as well as the noise level received inside has an order of magnitude lower than that received outside, thereby increase the SNR.

Table 9 represent the first resonance modes with each peak value of the tank measured inside and outside the tank. In this case, the first TE mode has higher power inside the tank than outside, while the first TM mode is lower inside, as expected. This trend it is not comply with the expectations for the TE mode. It is because the power takes very low values in the order of 10${}^{-8}$ $V^{2}$ and the measurement error is significant.

**Table 9:** Peak values for first TE and TM modes inside and outside the tank for surface PD on insulator.

Modes	Power ($V^{2}$)		
	Inside	Outside	Ratio
${\mathrm{TE}}_{011}$,${\mathrm{TE}}_{101}$ = 560 MHz	7.5 × $10^{-8}$	3.3 × $10^{-8}$	2.3
${\mathrm{TM}}_{110}$ = 655 MHz	4.8 × $10^{-8}$	8.8 × $10^{-8}$	0.5

Table 10 shows the cumulative power for the spectrum calculated inside and outside the tank in three representatives bands for this test object: up to 300 MHz, wherein the PD power decays; from 300 MHz to 1200 MHz, where the first resonances are; and 1200–2500 MHz band in which PD have low power. Inside the tank, most of the power received is located up to 300 MHz, and it is noteworthy that the tank amplifies the value of the power in this band. This can be attributed to the ground system of the enclosure of the model tank, which gives a poor RF shielding comparing with the grounding in a real power transformer. In the band 300–1200 MHz, the SNR has the same order of magnitude for both, inside and outside, however it is greater the power of the PD received from outside, as expected. Finally, the power contained in the last interval is approximately equal from both sensors.

**Table 10:** Accumulated power and SNR for surface PD in insulator over the tank when measured with the inner and the outer 10 cm monopoles.

Antenna	Power ($V^{2}$)		
	12 kV	0 V	SNR
Inside ∼300 MHz	7.1 × $10^{-6}$	4.1 × $10^{-8}$	171.5
Outside ∼300 MHz	4.8 × $10^{-6}$	3.0 × $10^{-7}$	16
Inside 300–1200 MHz	2.9 × $10^{-6}$	1.1 × $10^{-7}$	27.1
Outside 300–1200 MHz	6.7 × $10^{-6}$	2.1 × $10^{-7}$	32.3
Inside 1200–2500 MHz	6.2 × $10^{-7}$	2.8 × $10^{-7}$	2.2
Outside 1200–2500 MHz	4.2 × $10^{-7}$	2.4 × $10^{-7}$	1.7

## 7. Discussion

**Figure 13:**
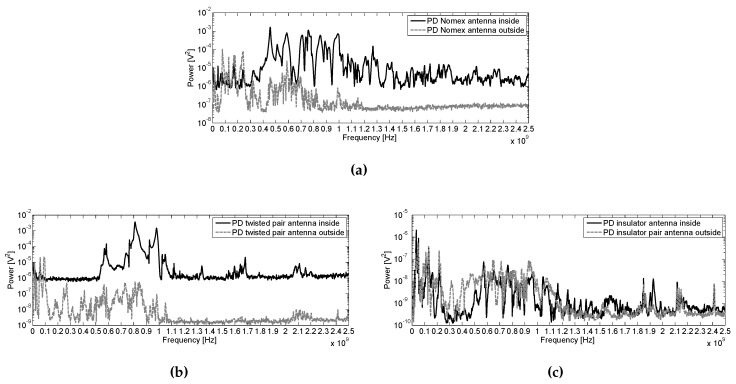
Average power spectra for PD measured from inside and outside the tank in (**a**) Nomex, (**b**) twisted pair and (**c**) insulator.

Though in the previous section the important findings when comparing the spectrum of the three tests samples are mentioned, it is important to state any information that is found as a result comparing their spectra and SNRs at different frequencies. Thus, in Figure 13 are compared the PD spectra inside and outside the tank for the three test objects. The base values for the PD spectra inside for Nomex, Figure 13a and for the twisted pair Figure 13b is clearly lower than from outside. However, in the insulator case, Figure 13c, the spectrum takes similar shape and base magnitudes.

Table 11 shows the comparison of SNR for the three test objects in three different frequency bands: up to 500 MHz, 500–1200 MHz and 1200–2500 MHz. For the first band, the SNR is clearly greater than 1 for the three samples and from inside and outside expect inside the tank for the twisted pair, due to the PD pulses have great power than this effect attributable to the set-up. In the 500–1200 MHz band, where the first resonances are, it is shown that it is possible to measure this frequency components from both sides of the tank enclosure. Finally, the 1200–2500 MHz band is mitigated outside the enclosure with SNR closers to unit.

**Table 11:** SNRs for the three test objects measured from inside and outside.

Antenna	Object	SNR
	Nomex	51.5
Inside ∼500 MHz	Twisted pair	1.2
	Insulator	127
	Nomex	131
Outside ∼500 MHz	Twisted pair	186.3
	Insulator	17.4
	Nomex	204
Inside 500–1200 MHz	Twisted pair	158.6
	Insulator	30.8
	Nomex	13.7
Outside 500–1200 MHz	Twisted pair	29.1
	Insulator	27.8
	Nomex	6.48
Inside 1200–2500 MHz	Twisted pair	1.7
	Insulator	2.04
	Nomex	1.06
Outside 1200–2500 MHz	Twisted pair	1.3
	Insulator	1.38

In real power transformers, one of the most important problems to carry out PD detection with the UHF method is the necessity to locate a sensor inside the tank or in dielectric windows of its enclosure, such as the oil drain valve. These necessities are very problematic and usually require switching-off the electrical asset and remove some part of oil. Besides, real transformers have a star point connected to the neutral that is piped outside the tank through the bushing to avoid the galvanic connection between the active part of the windings and the tank. Thus, the tank shields the impulses. However, the model tank used in this work does not have a real grounded system that made the tank be really like a screen for the pulses. Thus, the model tank acts as an emitter at VHF and the outer antenna can register its effect.

## 8. Conclusions

Partial discharge measurements performed inside the model of a transformer tank show that the spectrum of the received signals are influenced by several factors. The first is the cavity, which depending on its dimensions allowing a certain resonant modes that have power for certain frequencies. The second is the frequency response of the antenna. The third depends on the PD type. When the EM emission comes out, the tank acts as a low-pass filter with a cut-off frequency of about 1.2 GHz, so that the outer antenna is not able to receive power from the PD above this frequency. Furthermore, it was found that a higher power content may appear outside than inside, below 300 MHz, and this is attributed to the ground cable of the enclosure which behaves as an antenna that amplify the PD radiation below 300 MHz. When the insulator is located outside the tank, similar power below 300 MHz is measured inside than outside, this could be due to the insufficient EM enclosure of the tank model. In this case, similarly than with the other test objects, resonance modes of the tank are excite and the cumulative power has the same order of magnitude both inside and outside in the 500–1200 MHz range. These results suggest that taking into account only the power spectrum of the PD, it is not trivial to identify the kind of PD source and additional studies in future must be carried out. In this sense, it will be intended to have a voltage reference to represent the PRPD pattern to identify the type of source in each case. However, this requires the use of another hardware to carry out the measurements in order to capture both, fast pulses in the order of *ns*, such as PD, synchronizing with the AC voltage reference in the order of *ms*, so a synchronized acquisition system with uncouple channels, with different frequency sample will be required. In future research, it would be necessary to deploy artificial defects with similar PRPD distributions as those described in this work in a real power transformer to measure its PD activity in UHF with various sensor arrangements. Future research will be a step forward in this research area and will allow for applying the knowledge acquired in this paper for application in the field.
